# Altered subcortical and cortical brain morphology in adult women with 47,XXX: a 7-Tesla magnetic resonance imaging study

**DOI:** 10.1186/s11689-022-09425-1

**Published:** 2022-02-23

**Authors:** Chaira Serrarens, Maarten Otter, Bea C. M. Campforts, Constance T. R. M. Stumpel, Henk Jansma, Thérèse A. M. J. van Amelsvoort, Claudia Vingerhoets

**Affiliations:** 1 Department of Psychiatry and Neuropsychology, School for Mental Health and Neuroscience, Maastricht University, Maastricht, The Netherlands; 2 Medical Department, SIZA, Arnhem, The Netherlands; 3 Department of Community Mental Health in Mild Intellectual Disabilities, Trajectum, Zutphen, The Netherlands; 4 Department of Clinical Genetics and School for Oncology and Developmental Biology, Maastricht University Medical Centre, Maastricht, The Netherlands; 5 Department of Cognitive Neuroscience, Faculty of Psychology and Neuroscience, Maastricht University, Maastricht, The Netherlands; 6 Heeren Loo Zorggroep, Amersfoort, The Netherlands; 7 Department of Radiology and Nuclear Medicine, Amsterdam University Medical Centre, Amsterdam, The Netherlands

**Keywords:** 47,XXX, Adults, 7T, Subcortical volume, Cortical surface area, Cortical thickness, Cortical folding, Social functioning, Social cognition

## Abstract

**Background:**

Triple X syndrome (47,XXX) is a relatively common sex chromosomal aneuploidy characterized by the presence of a supernumerary X chromosome in females and has been associated with a variable cognitive, behavioural and psychiatric phenotype. 47,XXX may serve as a suitable model for studying the effect of genetic architecture on brain morphology. Previous studies have shown alterations in brain structure in 47,XXX particularly in childhood and adolescence. In this study, we examined subcortical and cortical brain morphology in adult women with 47,XXX using ultra-high field 7T MRI. Given previous evidence of impaired social functioning and emotion recognition in adults with 47,XXX, we also investigated the relationship of these functions with brain morphology.

**Methods:**

Twenty-one adult women with 47,XXX and 22 age- and sex-matched healthy controls were included. Structural T1-weighted images were acquired using a 7-Tesla magnetic resonance scanner. Measures of subcortical brain volumes, cortical surface area and thickness, and cortical folding were obtained and compared between the groups using general linear models. Additionally, we examined potential relationships between brain outcome measures and social functioning and social cognition in 47,XXX using correlation analyses.

**Results:**

Adults with 47,XXX showed lower volumes of the thalamus, caudate, putamen, hippocampus, nucleus accumbens and pallidum, and larger lateral ventricle volumes. Lower surface area was found in the superior frontal gyrus and superior temporal gyrus in 47,XXX participants compared to healthy controls. Altered cortical thickness and cortical folding were not present in 47,XXX. Cortical thickness was associated with social cognition in 47,XXX.

**Conclusions:**

Results suggest that a supernumerary X chromosome in females affects subcortical and lateral ventricle volumes, and cortical surface area in adulthood. 47,XXX may serve as a suitable model for studying genetic influences on structural brain morphology across developmental stages in order to understand neurobiological mechanisms underlying cognitive and behavioural impairments.

**Supplementary Information:**

The online version contains supplementary material available at 10.1186/s11689-022-09425-1.

## Background

Triple X syndrome (47,XXX) is a relatively common sex chromosomal aneuploidy (SCA) characterized by the presence of a supernumerary X chromosome in females with an estimated incidence of about one in 1000 female newborns [1]. Individuals with 47,XXX display relatively subtle physical characteristics and present with a variable behavioural and cognitive profile, ranging from severe impairments to average or above average functioning [2]. Therefore, many affected females do not have a distinct appearance and may go undiagnosed. However, children with 47,XXX are at increased risk for developing language difficulties [3], show delays in motor development [1] and display problems in forming good interpersonal relationships [2]. Furthermore, individuals with 47,XXX are at increased risk for neurodevelopmental disorders, such as attention deficit hyperactivity disorder (ADHD) and autism spectrum disorder (ASD) [4]. In addition, mild learning disabilities and cognitive impairments including deficits in inhibition, mental flexibility, sustained attention and (visual) working memory have been reported in children and adolescents with 47,XXX [2, 5, 6]. Many girls with 47,XXX display deficits across both verbal and performance IQ, with verbal IQ more affected [1]. In addition, social functioning and social cognition deficits have been described in children [7–9] and recently also in adults with 47,XXX [10]. Furthermore, psychiatric disorders including anxiety and depression are more prevalent in women with 47,XXX compared to the general population [11].

While most studies focus on the cognitive, behavioural and psychiatric phenotype of 47,XXX, less is known about the neurobiological effects of a supernumerary X chromosome, including effects on brain morphology. In the last two decades, SCAs including Turner and Klinefelter syndrome, and 47,XXX, have received increased attention since they serve as promising models for examining the effects of sex chromosomes on brain development and on neurodevelopmental disorders. While most neuroimaging studies using structural magnetic resonance imaging (sMRI) to study brain structure focused on children and adolescents with 47,XXX, neuroimaging studies in adult 47,XXX women are still scarce. Therefore, it remains unclear whether differences in brain morphology in 47,XXX remain static across developmental stages. Several sMRI studies have investigated the effect of a supernumerary X chromosome on global brain anatomy. Individuals with 47,XXX showed a reduction in total brain volume compared to female controls in childhood and adolescence [12, 13]. Moreover, when sMRI data between children and adolescents with a variety of SCAs and typically developing females and males were compared, an association between X chromosome supernumeracy and a decrease in total brain volume and total cortical volume was shown [14]. Consistent with findings in 47,XXX children and adolescents, adults with 47,XXX showed a reduction in total brain volume [13, 15]. Differences in regional brain anatomy have also been observed between 47,XXX and controls. Analyses in a SCA population revealed that in childhood and adolescence, carriage of a supernumerary X chromosome was associated with decreased amygdala volumes compared to gonadally matched controls (i.e. 47,XXX vs 46,XX and other SCAs vs 46,XY), also when adjusting for total brain volume [16]. Furthermore, this study showed that in childhood and adolescence, a supernumerary X chromosome was associated with decreased hippocampal volumes relative to gonadally matched controls, although the effect was not present when adjusting for total brain volume using an allometric method. Another study investigating SCA effects on subcortical volume in childhood and adolescence showed that SCAs, including 47,XXX, were associated with large-effect size reductions in striatal, thalamic and pallidum volume exhibiting the largest effect sizes for pallidum volume loss [17]. Lenroot et al. [12] found that in childhood and adolescence, an additional X chromosome in females is associated with decreased grey and white matter brain volumes in all brain regions except the parietal lobe. Additionally, this study showed areas of both increased and decreased cortical thickness in children and adolescents with 47,XXX. Thicker cortex was found bilaterally in the medial prefrontal and medial temporal lobes, regions that, according to the authors, could be related to difficulties with social interactions and anxiety symptoms. Thinner cortex was found in both lateral temporal lobes, which are potentially related to the language problems frequently observed in 47,XXX. Conversely, X chromosome supernumeracy in childhood and adolescence was shown to be predominantly associated with a loss of cortical surface area while leaving cortical thickness relatively unaltered [14]. Significant local cortical surface area reductions were found in bilateral frontotemporal cortices which were associated with increasing X chromosome dosage. Another study in children and adolescents investigating the effect of sex chromosomes on human cortical folding — or gyrification/sulcation — showed that supernumerary X chromosomes (i.e. XXX vs XX and XXY vs XY) were associated with reductions in all folding metrics, with greater reductions in total brain area, hull and sulcal area, and sulcal length than for the sulcal index (ratio between total sulcal surface area and hull area) and sulcal depth [18]. Lastly, when investigating amygdala and hippocampal volumes in adult women with 47,XXX and matched control women, a trend was shown for reduced amygdala volume in 47,XXX [15].

Overall, these findings suggest alterations of brain structure in 47,XXX particularly in childhood and adolescence. However, our understanding of the effect of an extra X chromosome in adult women on cortical and subcortical morphology remains incomplete, due to the small number of studies that have been carried out. In addition, the majority of studies on brain structure in 47,XXX focused on a single brain outcome measure and have been performed at a standard field strength of 1.5 Tesla, which is known to have lower spatial resolution compared to ultra-high field MRI. Therefore, the aim of the present study was to compare multiple levels of brain morphology including subcortical brain volumes, cortical surface area and thickness, as well as cortical folding between adult women with 47,XXX and sex-matched healthy controls using ultra-high field 7T MRI data. Given previous evidence of impaired social functioning and cognition in adults with 47,XXX, we also examined potential relationships between brain outcome measures and social functioning and emotion recognition in adults with 47,XXX.

## Methods

All procedures in this study were performed in accordance with the ethical standards established by the respective national and institutional committees regarding human experimentation and in accordance with the Declaration of Helsinki. In addition, all procedures involving human subjects were approved by the Medical Ethics Committee of the Maastricht University Medical Centre, Maastricht, the Netherlands (METC143051/NL46871.068.14). Written informed consent was obtained from all participants.

### Participants

Twenty-one adult women with 47,XXX and 22 age- and sex-matched healthy controls, aged 18–59, were included in this study. The Dutch (NL) and Flemish (B) individuals with 47,XXX were recruited through the 47,XXX support group, clinicians, clinical geneticists, paediatricians and gynaecologists. Healthy controls were recruited independently through local advertisement. General inclusion criteria were (1) 18 years or older of age, (2) mental capacity to give informed consent and (3) a sufficient command of the Dutch language. 47,XXX women were included on the basis of a confirmed 47,XXX karyotype or a mosaic 46,XX/47,XXX karyotype with at least 85% cells with an extra X chromosome. Exclusion criteria for all study participants were (1) being under legal guardianship, (2) contraindications for MRI and (3) pregnancy.

### Instruments

The full-scale intelligence quotient (FSIQ) of all study participants was assessed using the shortened version of the Dutch Wechsler Adult Intelligence Scale, Third Edition (WAIS-III; [19]). The Emotion Recognition Task (ERT) of the Cambridge Neuropsychological Test Automated Battery (CANTAB; Cambridge Cognition, Cambridge, UK; see www.cantab.com) was used to assess social cognition in all participants. A Dutch translation of the informant/observer version of the Social Responsiveness Scale for adults (SRS-A) was used to assess social behaviours associated with ASD in all participants [20]. The SRS-A questionnaire is subdivided into 4 subscales including (1) social awareness, (2) social communication, (3) social motivation and (4) rigidity and repetitive behaviour. SRS-A scales are reported as T-scores with scores < 40 indicating high social functioning, scores between 40 and 59 indicating normal social functioning, scores between 60 and 75 indicating mild to moderate social deficits and scores ≥ 76 indicating severe deficits.

### Structural MR data acquisition

Whole-brain MRI acquisition took place at the Scannexus brain imaging centre (https://scannexus.nl) and was performed on a MAGNETOM 7T MR scanner (Siemens Healthineers, Erlangen, Germany) using a single-channel transmit/32-channel receive head coil (Nova Medical Inc., Wilmington, MA, USA). The anatomical (T1-weighted) image was acquired using the magnetization-prepared 2 rapid acquisition gradient-echo (MP2RAGE) sequence; repetition time (TR) = 5000 ms; echo time (TE) =2.51 ms; inversion times TI1/TI2 = 900/2750 ms; flip angle = 5°/3°; phase partial Fourier = 6/8; GRAPPA factor = 2 with 24 reference lines; bandwidth = 248 Hz/Px; voxel size = 0.7 × 0.7 × 0.7 mm; and acquisition time = 10:57 min.

### Procedure

This study was a cross-sectional cohort study conducted at the Scannexus brain imaging centre (https://scannexus.nl). On the testing day, study procedures were again explained to the participants. To measure brain structure, participants underwent one structural magnetic resonance imaging measurement. On the same day, the ERT of the CANTAB and the shortened version of the WAIS-III were conducted. Relatives of study participants were requested to fill in the SRS-A questionnaire.

### Image processing

First, background noise of T1-weighted images was suppressed to improve the accuracy of segmentation. Cortical reconstruction and volumetric segmentation were performed using the fully automated and validated segmentation software FreeSurfer, version 6 (https://surfer.nmr.mgh.harvard.edu/; [21]). FreeSurfer performs motion correction, Talairach transformation, removal of non-brain tissue, segmentation of cortical regions and subcortical structures, intensity normalization and cortical reconstruction. Subcortical structures and cortical regions were visually inspected to assess quality. Using FreeSurfer, manual editing of the putamen was performed for a few subjects because of poor quality segmentations. Using probabilistic information from a manually labelled training set, each voxel of volume was automatically assigned to neuroanatomical labels. Volumes for eight bilateral regions of interest were derived, including lateral ventricles, nucleus accumbens, amygdala, caudate, hippocampus, putamen, pallidum and thalamus along with intracranial volume (ICV). The cerebral cortex of each T1-weighted image was parcellated into 68 (34 per hemisphere) cortical grey matter regions according to the Desikan-Killiany atlas [22]. Measures of cortical surface area and cortical thickness for each cortical region, as well as two whole-hemisphere measures, were obtained. The degree of cortical folding was measured by the local gyrification index (LGI) according to the method of Schaer et al. [23], implemented in FreeSurfer. LGI is a metric that quantifies the amount of cortex buried within the sulcal folds as compared with the amount of cortex on the outer visible cortex in circular regions of interest. Average LGI values were extracted from the 34 gyral regions of interest for each hemisphere.

### Statistical analyses

All statistical analyses were performed in R, version 3 [24]. Differences in group demographics including age and FSIQ were examined using Mann-Whitney *U*-tests and independent samples *t*-tests according to the normality of data distribution.

Group differences in subcortical volume, cortical thickness and surface area, and cortical folding were examined using general linear models (GLM) via the *lm* function in R, with each brain outcome measure as the dependent variable and group (i.e. diagnosis) as the independent variable, adjusted for FSIQ. Furthermore, we included ICV as covariate in analyses with subcortical volume, surface area and cortical folding. Given that head size does not scale with cortical thickness, ICV was not included as a covariate in the cortical thickness analyses [25]. Cohen’s *d* effect size estimates were derived from the t-statistic of the group variable from the GLM. *P*-values were corrected for multiple comparisons using the Benjamini-Hochberg procedure [26] to ensure a false-discovery rate (FDR) limited at 5% (*q* = 0.05) for either 17 measures (subcortical volume regions of interest), 70 measures (cortical thickness or surface area regions of interest) or 68 measures (cortical folding regions of interest). Results were considered significant if the FDR corrected *p*-value was < .05.

In addition, we examined whether brain outcome measures were associated with social cognition and social functioning. Normally distributed ERT scores were compared between groups using the independent samples *t*-test. Correlations between ERT scores and brain measures were computed using Pearson’s correlation coefficients, separately for 47,XXX and healthy controls. Normally and non-normally distributed total SRS-A T-scores and T-scores for SRS-A subscales were compared using independent samples *t*-tests and Mann-Whitney *U*-tests, respectively. Pearson and Spearman’s rank correlation coefficients were used to compute the relationship between brain outcome measures and SRS-A T-scores, separately for 47,XXX and healthy controls. All correlation analyses included correction for multiple comparisons using the procedure described above.

## Results

### Demographic characteristics

Demographic characteristics for 47,XXX and healthy control subjects are presented in Table 1. There was no significant difference in age between groups. Women with 47,XXX had a significantly lower FSIQ compared to healthy controls.

**Table 1:** Sample demographics

	47,XXX ***N*** = 21 *Mean (SD)*	Healthy controls ***N*** = 22 *Mean (SD)*	Statistic	***p***-value
Age	30.14 (11.84)	33.86 (12.43)	*U* = 276	0.279
FSIQ	85.81(10.44)	99.73 (12.32)	*t* = 3.99	< .001*

*FSIQ:* full-scale intelligence quotient; *p-value < .05

### Social cognition and social functioning

ERT and SRS-A T-scores are summarized in Table 2. ERT scores were significantly lower in 47,XXX subjects compared to healthy controls. Women with 47,XXX had significantly higher scores on 3 SRS-A subscales: social awareness, social communication and social motivation, as well as on total SRS-A score. There was no significant difference between groups in score of SRS-A subscale rigidity and repetitive behaviour.

**Table 2:** Summary of ERT and SRS-A T-scores

		47,XXX *Mean (SD)*		Healthy controls *Mean (SD)*	Statistic	***p***-value
Social cognition^a^	*N*		*N*			
ERT score	21	101.33 (17.56)	22	119.59 (14.05)	*t* = 3.77	< .001*
Social functioning^b^						
Social awareness score	20	56.85 (10.73)	22	49.36 (11.24)	*t* = −2.20	0.033*
Social communication score	20	55.85 (7.89)	22	46.86 (7.82)	*t* = −3.71	< .001*
Social motivation score	20	54.75 (8.08)	22	45.64 (7.34)	*U* = 87.5	< .001*
Rigidity and repetitive behaviour score	20	53.50 (9.70)	22	48.86 (10.45)	*U* = 152.5	0.090
Total score	20	56.05 (8.02)	22	47.32 (9.47)	*t* = −3.21	0.003*

^a ^Measured using the CANTAB. ^b ^Measured using the SRS-A questionnaire. *ERT:* emotion recognition task; *p-value < .05

### Subcortical brain morphology

**Fig. 1:**
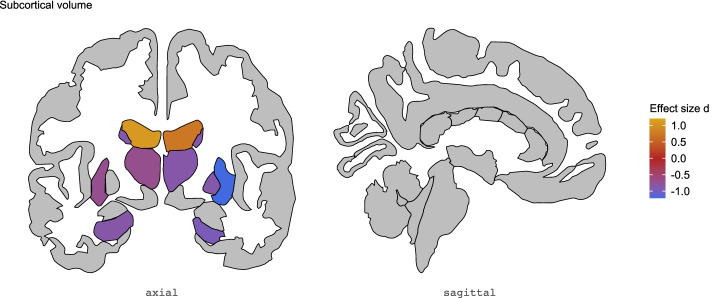
Subcortical volume differences between 47,XXX women and healthy controls. Effect sizes for significant differences in volumes of subcortical brain regions between 47,XXX subjects and healthy controls. Negative effect sizes indicate lower subcortical brain volume in 47,XXX subjects compared to healthy controls

Analyses revealed significant group differences in volumes across the majority of subcortical regions of interest (12 out of 17) with large effect sizes (Cohen's* d* ranging between −1.197 and 1.073; Table 3 and Fig. 1). Significantly lower volumes of the bilateral thalamus, caudate, putamen and hippocampus, as well as lower volume of the left nucleus accumbens and right pallidum, were observed in 47,XXX subjects compared to healthy controls. In addition, 47,XXX subjects showed significantly higher bilateral lateral ventricle volumes compared to healthy controls.

**Table 3:** Results for volume of each subcortical region of interest for the 47,XXX subjects versus healthy controls comparison controlling for FSIQ and ICV

	Cohen’s *d* (47,XXX — HC)	Standard error	95 % CI	FDR *p*-value
ICV	−0.197	0.306	−0.797–0.402	0.521
Left accumbens	−0.873	0.320	−1.500 to −0.246	0.015*
Left amygdala	−0.381	0.308	−0.985–0.222	0.248
Left caudate	−0.888	0.320	−1.516 to −0.260	0.015*
Left hippocampus	−0.854	0.319	−1.480 to −0.229	0.015*
Left lateral ventricle	1.073	0.327	0.431–1.714	0.010*
Left pallidum	−0.481	0.310	−1.088–0.126	0.161
Left putamen	−0.695	0.315	−1.312 to −0.079	0.040*
Left thalamus	−0.694	0.315	−1.310 to −0.077	0.040*
Right accumbens	−0.451	0.309	−1.057–0.155	0.179
Right amygdala	−0.354	0.308	−0.957–0.249	0.268
Right caudate	−0.853	0.319	−1.479 to −0.227	0.015*
Right hippocampus	−0.954	0.323	−1.587 to −0.322	0.015*
Right lateral ventricle	0.727	0.315	0.108–1.345	0.038*
Right pallidum	−0.868	0.320	−1.494 to −0.241	0.015*
Right putamen	−1.197	0.332	−1.848 to −0.545	0.006*
Right thalamus	−0.856	0.319	−1.482 to −0.230	0.015*

*HC:* healthy controls; *CI:* confidence interval; *ICV:* intracranial volume; *FDR:* false-discovery rate; *FDR *p*-value < .05

### Cortical brain morphology

**Fig. 2:**
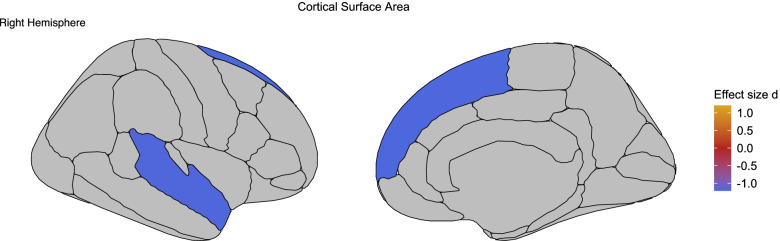
Cortical surface area differences between 47,XXX women and healthy controls. Effect sizes for significant differences in surface area of cortical brain regions between 47,XXX subjects and healthy controls. Negative effect sizes indicate lower cortical surface area in 47,XXX subjects compared to healthy controls

Case-control surface area differences for all cortical structures are listed in Supplementary Table 1. Significantly lower surface area was found in the superior frontal gyrus (Cohen's* d* = −1.161) and superior temporal gyrus (Cohen's* d =* −1.171) of the right hemisphere in 47,XXX subjects compared to healthy controls (Fig. 2). No significant differences in cortical thickness and cortical folding regions were found between 47,XXX and healthy control subjects (Supplementary Tables 2 and 3).

### Relationships with social cognition and social functioning in 47,XXX

**Fig. 3:**
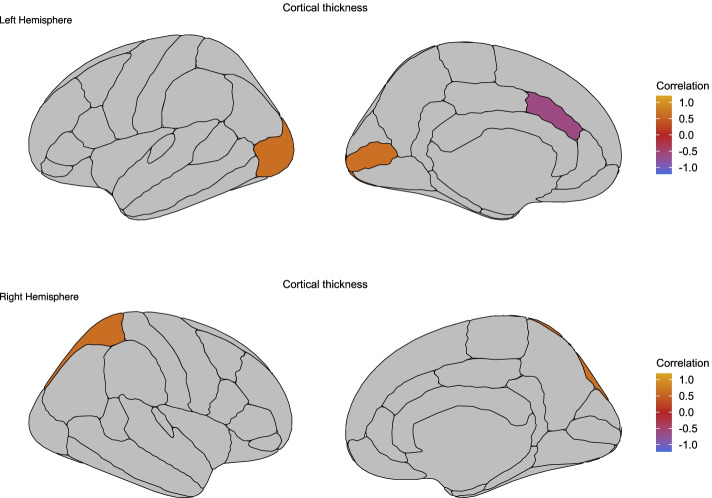
Correlation between cortical thickness regions and social cognition in 47,XXX. Correlations for significant relationships between cortical thickness regions and ERT scores in 47,XXX subjects

Within the 47,XXX group, thickness of 3 out of 70 cortical regions of interest, including the left lateral occipital cortex, left pericalcarine cortex and right superior parietal cortex, was positively correlated with social cognition (*r* between 0.639 and 0.646; Fig. 3; Supplementary Table 4). In contrast, 47,XXX subjects showed a negative correlation between cortical thickness of the left caudal anterior cingulate cortex and social cognition (*r* = −0.618; Fig. 3). We found no significant correlations between thickness of cortical regions and SRS-A scores. In addition, we found no significant correlations between other brain outcome measures and social cognition, as well as with SRS-A scores (all FDR *p*-value > .05). Lastly, significant correlations between brain outcome measures and behavioural measures were not present in healthy controls (all FDR *p*-value > .05).

## Discussion

This is the first ultra-high field MRI study in adults with 47,XXX investigating multiple levels of brain morphology, including subcortical volume, cortical thickness and surface area, and cortical folding. We demonstrated that in adulthood, the presence of a supernumerary X chromosome in females affects subcortical and lateral ventricle volumes as well as cortical surface area, whereas alterations in cortical thickness and cortical folding were not present. However, cortical thickness was associated with social cognition in 47,XXX.

Our findings of smaller subcortical volumes and larger lateral ventricle volumes in adults with 47,XXX (medium to high effect sizes) are largely in line with those of previous cross-sectional 1.5T neuroimaging studies in children and adolescents [12, 17]. This possibly suggests that carriage of a supernumerary X chromosome in women exerts similar effects on volumetric alterations of lateral ventricles, striatum, pallidum and thalamus across neurodevelopmental stages. In opposition to the findings of a previous study conducted in 47,XXX adults [15], we showed reduced hippocampal volumes in 47,XXX during adulthood. The hippocampus is a key element of a limbic brain network that is disturbed in several neuropsychiatric disorders [27] and autism [28]. Analyses in a SCA population comprised of children and adolescents did not show a significant association between X chromosome supernumeracy and decreased hippocampal volumes when adjusting for total brain volume using allometric scaling, a technique which was not implemented in our analyses since ICV did not significantly differ between groups [16]. This allometric method allows quantification of non-linear scaling relationships between regional and total brain volume using data from separate, typically developing participants. In addition, the studies of Patwardhan et al. [15] and Nadig et al. [16] report 1.5T MRI data, which could possibly have led to difficulties in properly detecting the hippocampal — amygdala border. 7T MRI is known to have higher sensitivity to tissue changes and anatomical details, which thus produces higher spatial resolution and clearer tissue boundaries [29]. Interestingly, we did not show differences between groups in amygdala volume, which is also a key element of the limbic brain network that plays a central role in emotional and social information processing, is involved in complex social judgements and is part of a neural network underlying social cognition [30]. Since social impairments have frequently been reported in 47,XXX, we expected to find alterations in amygdala volume in our study. A previous study by Patwardhan et al. [15] in adults with 47,XXX also reported unaltered amygdala volumes in a small sample of 47,XXX women (*N* = 10). Combined, these results suggest that the amygdala is unaffected in 47,XXX during adulthood. However, smaller amygdala volumes were found by Nadig et al. [16] in children and adolescents with 47,XXX compared to gonadally matched controls. It could be hypothesized that discrepancies between study findings concerning alterations in amygdala and hippocampal volumes relate to the developmental status of participants. Although we found significant differences in social cognition and social functioning between 47,XXX women and controls, social responsiveness scores in 47,XXX indicated normal social functioning. Since the amygdala is strongly involved in social cognition and social behaviour, it could be hypothesized that lower amygdala volumes in 47,XXX children reported by Nadig et al. [16] may have been related to more impairments in social cognition and social behaviour in their sample. However, these measures were not included in their study.

In addition, we report lower surface area in the superior temporal gyrus and the superior frontal gyrus of the right hemisphere in adults with 47,XXX, showing high effect sizes. These findings are in line with a previous study performed in children and adolescents showing associations between surface area reductions in frontotemporal cortices and greater X chromosome dosage. The superior temporal gyrus has been involved in auditory processing, including language, and has been implicated as a critical structure in social cognition [31]. Yet, a correlation between surface area of the superior temporal gyrus and social cognition in 47,XXX was not present in our study. Lower superior temporal gyrus surface area might be also related to language problems often observed in 47,XXX. Since linguistic function was not assessed in our study, this possible relationship needs further investigation. The superior frontal gyrus is involved in higher cognitive functions, particularly in working memory [32]. Future studies could address the possible relationship between the superior frontal gyrus and working memory in 47,XXX.

We showed cortical differences between 47,XXX and healthy controls specifically in surface area. Differences in cortical thickness areas and cortical folding between groups were not present. Contrary, differences in cortical thickness were previously reported in 47,XXX [12]. In addition, differences in cortical folding were reported in childhood and adolescence in individuals with a supernumerary X chromosome relative to gonadally matched controls [18]. To date, the exact processes driving age-related changes on different dimensions of the cerebral cortex, including on surface area, cortical thickness and cortical folding and their relationship with cognitive and behavioural impairments, remain unclear. The primary process driving cortical surface area is cortical column generation [33]. This process might be sensitive to X chromosome effects, resulting in cortical surface area differences in 47,XXX in childhood that remain observable in adolescence and adulthood. The primary process driving cortical thickness is the genesis of neurons [33], and previous research in healthy subjects found an inverse relation between cortical thickness and neuronal density [34]. Cortical thickness increase or decrease observed in 47,XXX children and adolescents might be driven by a reduction or increase in neuron number, respectively. We could hypothesize that these alterations in neuron numbers normalize towards adulthood in 47,XXX, resulting in no observable differences in cortical thickness compared to healthy controls. Lastly, multiple theories of cortical folding exist that are trying to explain the mechanisms that drive gyrification [35]. However, the exact mechanisms remain to be elucidated. We could hypothesize that X chromosome supernumeracy in females alters or delays cortical folding maturation, resulting in differences in earlier developmental stages that are eventually diminished during adulthood. Future longitudinal studies are necessary to gain more insight in these processes. Since 47,XXX has been associated with cognitive impairments and behavioural characteristics of varying severity, the lack of differences in cortical thickness and cortical folding between groups could also be related to less severe social, cognitive and language impairments in our sample. For example, Lenroot et al. [12] reports a high incidence of language problems and social phobia in their sample, which could possibly explain discrepancies in cortical thickness results between studies.

Finally, exploratory analyses showed correlations between cortical thickness regions and social cognition in 47,XXX, although the underlying mechanisms are not fully understood. Yet, a previous meta-analysis of fMRI studies by Fusar-Poli et al. [36] showed that processing of emotional faces was associated with increased activation in a number of visual areas (including the occipital gyri), limbic areas (including the cingulate cortex) and temporoparietal areas (including the parietal lobe). The left lateral occipital cortex, the left pericalcarine cortex (component of the occipital lobe), the left caudal anterior cingulate cortex and the right superior parietal cortex, areas that showed increased activation to emotional faces in the fMRI meta-analysis by Fusar-Poli [36], showed a significant relationship with the emotion recognition task performed in our study. Correlations with social cognition were not present for other brain measures. Moreover, we did not show correlations between social functioning and brain measures in women with 47,XXX despite significant differences in social cognition and social functioning between 47,XXX and healthy controls.

Future studies examining effects of brain alterations on social cognition and social functioning in 47,XXX are warranted.

### Strengths and limitations

This study has both strengths and limitations. Our study is the first to investigate brain morphology of individuals with 47,XXX using ultra-high field 7T MRI data. There are several advantages of 7T MRI compared to conventional field strength imaging such as 1.5T and 3T MRI. The main advantage of 7T MRI is its increased sensitivity. The resulting increased signal-to-noise ratio (SNR) allows imaging with a higher spatial resolution, improves the delineation of anatomical structures, as well as subtle pathology, and provides clearer tissue boundaries which results in improved segmentation accuracy [29, 37]. Second, our study is one of the first studies examining both subcortical and cortical brain morphology in 47,XXX during adulthood. Moreover, our study is the first to examine relationships between brain outcome measures and social functioning and social cognition in 47,XXX. Our findings should also be considered in light of certain limitations. First, our sample size was relatively small compared to previous studies examining brain morphology in a variety of SCAs including 47,XXX, resulting in decreased power to detect statistically significant differences. Second, the cross-sectional nature of our data makes it difficult to assess how observed effects are patterned across developmental trajectories in 47,XXX individuals, stressing the need for longitudinal studies investigating brain morphology in 47,XXX. Third, although healthy control women were screened for phenotypical traits often observed in 47,XXX, the absence of 47,XXX was not genetically verified. Therefore, we cannot completely rule out the absence of 47,XXX in healthy controls. Lastly, we did not control for ascertainment bias, which is a well-known limitation in 47,XXX studies as patients presenting more severe phenotypes are more likely to be clinically recognized and enrolled in research.

## Conclusions

In conclusion, our results indicate a key role of a supernumerary X chromosome on subcortical and cortical brain morphology in adult women. 47,XXX may serve as a suitable model for studying genetic influences across developmental stages on structural brain morphology in order to understand neurobiological mechanisms underlying cognitive and behavioural impairments.

## Supplementary Information

**Additional file 1: Supplementary Table S1.** Results for surface area of each cortical region of interest for the 47,XXX subjects versus healthy controls comparison controlling for FSIQ and ICV. **Supplementary Table S2.** Results for thickness of each cortical region of interest for the 47,XXX subjects versus healthy controls comparison controlling for FSIQ. **Supplementary Table S3.** Results for folding of each cortical region of interest for the 47,XXX subjects versus healthy controls comparison controlling for FSIQ and ICV. **Supplementary Table S4.** Correlation between thickness of each cortical region of interest and social cognition in 47,XXX subjects.

## Data Availability

The dataset used and analysed during the current study is available from the corresponding author on reasonable request.

## Abbreviations

47,XXX: Triple X syndrome
ADHD: Attention deficit hyperactivity disorder
ASD: Autism spectrum disorder
CANTAB: Cambridge Neuropsychological Test Automated Battery
ERT: Emotion recognition task
FDR: False-discovery rate
FSIQ: Full-scale intelligence quotient
GLM: General linear model
ICV: Intracranial volume
LGI: Local gyrification index
SCA: Sex chromosomal aneuploidy
sMRI: Structural magnetic resonance imaging
SRS-A: Social Responsiveness Scale for adults
WAIS-III: Wechsler Adult Intelligence Scale, Third Edition
