# Supplementary material for: Altered subcortical and cortical brain morphology in adult women with 47,XXX: a 7-Tesla magnetic resonance imaging study
Source: J Neurodev Disord. 2022 Feb 23;14:14. doi: 10.1186/s11689-022-09425-1 (PMC8903568; doi:10.1186/s11689-022-09425-1)
Supplement: Supplementary file 1 — Additional file 1: Supplementary Table S1. Results for surface area of each cortical region of interest for the 47,XXX subjects versus healthy controls comparison controlling for FSIQ and ICV. Supplementary Table S2. Results for thickness of each cortical region of interest for the 47,XXX subjects versus healthy controls comparison controlling for FSIQ. Supplementary Table S3. Results for folding of each cortical region of interest for the 47,XXX subjects versus healthy controls comparison controlling for FSIQ and ICV. Supplementary Table S4. Correlation between thickness of each cortical region of interest and social cognition in 47,XXX subjects. [file 11689_2022_9425_MOESM1_ESM.docx]

**Supplementary Tables**

**Content:**

- **Supplementary Table S1:** Results for surface area of each cortical region of interest for the 47,XXX subjects versus healthy controls comparison controlling for FSIQ and ICV.
- **Supplementary Table S2: ﻿**Results for thickness of each cortical region of interest for the 47,XXX subjects versus healthy controls comparison controlling for FSIQ.
- **Supplementary Table S3:** Results for folding of each cortical region of interest for the 47,XXX subjects versus healthy controls comparison controlling for FSIQ and ICV.
- **Supplementary Table S4:** Correlation between thickness of each cortical region of interest and social cognition in 47,XXX subjects.

**Table S1. ﻿**Results for surface area of each cortical region of interest for the 47,XXX subjects versus healthy controls comparison controlling for FSIQ and ICV.

|  | Cohen’s d  (47,XXX – HC) | Standard error | 95 % CI | FDR *p*-value |
| --- | --- | --- | --- | --- |
| Left banks superior temporal sulcus | -0.494 | 0.310 | -1.101 - 0.113 | 0.256 |
| Left caudal anterior cingulate cortex | -0.056 | 0.305 | -0.655 - 0.542 | 0.908 |
| Left caudal middle frontal gyrus | -0.202 | 0.306 | -0.802 - 0.397 | 0.631 |
| Left cuneus | 0.501 | 0.310 | -0.107 - 1.109 | 0.253 |
| Left entorhinal cortex | -0.780 | 0.317 | -1.401 - -0.158 | 0.119 |
| Left frontal pole | -0.335 | 0.307 | -0.937 - 0.268 | 0.443 |
| Left fusiform gyrus | -0.688 | 0.314 | -1.304 - -0.072 | 0.142 |
| Left inferior parietal cortex | -0.310 | 0.307 | -0.911 - 0.292 | 0.462 |
| Left inferior temporal cortex | -0.584 | 0.312 | -1.195 - 0.027 | 0.176 |
| Left insula | -0.710 | 0.315 | -1.327 - -0.092 | 0.136 |
| Left isthmus cingulate cortex | -0.373 | 0.308 | -0.976 - 0.231 | 0.373 |
| Left lateral occipital cortex | 0.089 | 0.305 | -0.510 - 0.687 | 0.857 |
| Left lateral orbi-frontal cortex | -0.515 | 0.310 | -1.124 - 0.093 | 0.248 |
| Left lingual gyrus | 0.288 | 0.307 | -0.313 - 0.889 | 0.491 |
| Left medial orbitofrontal cortex | -0.133 | 0.305 | -0.732 - 0.466 | 0.751 |
| Left middle temporal gyrus | -0.284 | 0.307 | -0.885 - 0.317 | 0.491 |
| Left paracentral lobule | -0.137 | 0.305 | -0.736 - 0.461 | 0.751 |
| Left parahippocampal gyrus | 0.055 | 0.305 | -0.543 - 0.653 | 0.908 |
| Left pars opercularis | -0.400 | 0.308 | -1.004 - 0.205 | 0.355 |
| Left pars orbitalis | -0.469 | 0.309 | -1.075 - 0.138 | 0.273 |
| Left pars triangularis | -0.810 | 0.318 | -1.433 - -0.187 | 0.114 |
| Left pericalcarine cortex | 0.621 | 0.313 | 0.008 - 1.234 | 0.161 |
| Left postcentral gyrus | -0.084 | 0.305 | -0.683 - 0.514 | 0.857 |
| Left posterior cingulate cortex | -0.649 | 0.313 | -1.263 - -0.035 | 0.161 |
| Left precentral gyrus | -0.327 | 0.307 | -0.929 - 0.275 | 0.443 |
| Left precuneus | -0.020 | 0.305 | -0.618 - 0.578 | 0.947 |
| Left rostral anterior cingulate cortex | -0.212 | 0.306 | -0.812 - 0.388 | 0.625 |
| Left rostral middle frontal gyrus | -0.480 | 0.310 | -1.087 - 0.127 | 0.271 |
| Left superior frontal gyrus | -0.890 | 0.321 | -1.519 - -0.262 | 0.102 |
| Left superior parietal cortex | 0.186 | 0.306 | -0.413 - 0.785 | 0.658 |
| Left superior temporal gyrus | -0.685 | 0.314 | -1.301 - -0.069 | 0.142 |
| Left supramarginal gyrus | -0.549 | 0.311 | -1.159 - 0.060 | 0.214 |
| Left temporal pole | -0.426 | 0.309 | -1.031 - 0.179 | 0.332 |
| Left transverse temporal gyrus | -0.404 | 0.308 | -1.008 - 0.201 | 0.355 |
| Surface area left hemisphere | -0.628 | 0.313 | -1.241 - -0.015 | 0.161 |
| Right banks superior temporal sulcus | -0.473 | 0.310 | -1.079 - 0.134 | 0.273 |
| Right caudal anterior cingulate cortex | 0.146 | 0.306 | -0.453 - 0.745 | 0.740 |
| Right caudal middle frontal gyrus | -0.279 | 0.307 | -0.880 - 0.322 | 0.493 |
| Right cuneus | 0.737 | 0.316 | 0.119 - 1.356 | 0.119 |
| Right entorhinal cortex | -0.051 | 0.305 | -0.649 - 0.547 | 0.908 |
| Right frontal pole | -0.233 | 0.306 | -0.833 - 0.367 | 0.582 |
| Right fusiform gyrus | -0.810 | 0.318 | -1.433 - -0.187 | 0.114 |
| Right inferior parietal cortex | -0.927 | 0.322 | -1.558 - -0.296 | 0.099 |
| Right inferior temporal cortex | -0.313 | 0.307 | -0.915 - 0.289 | 0.462 |
| Right insula | -0.609 | 0.312 | -1.222 - 0.003 | 0.161 |
| Right isthmus cingulate cortex | -0.201 | 0.306 | -0.800 - 0.399 | 0.631 |
| Right lateral occipital cortex | -0.522 | 0.310 | -1.130 - 0.087 | 0.247 |
| Right lateral orbitofrontal cortex | -0.388 | 0.308 | -0.992 - 0.216 | 0.360 |
| Right lingual gyrus | 0.174 | 0.306 | -0.425 - 0.773 | 0.677 |
| Right medial orbi-frontal cortex | -0.297 | 0.307 | -0.898 - 0.304 | 0.480 |
| Right middle temporal gyrus | -0.586 | 0.312 | -1.198 - 0.025 | 0.176 |
| Right paracentral lobule | -0.624 | 0.313 | -1.237 - -0.011 | 0.161 |
| Right parahippocampal gyrus | -0.644 | 0.313 | -1.258 - -0.030 | 0.161 |
| Right pars opercularis | -0.746 | 0.316 | -1.366 - -0.127 | 0.119 |
| Right pars orbitalis | -0.761 | 0.316 | -1.381 - -0.141 | 0.119 |
| Right pars triangularis | -0.756 | 0.316 | -1.376 - -0.136 | 0.119 |
| Right pericalcarine cortex | 0.638 | 0.313 | 0.025 - 1.252 | 0.161 |
| Right postcentral gyrus | -0.274 | 0.307 | -0.875 - 0.327 | 0.495 |
| Right posterior cingulate cortex | -0.391 | 0.308 | -0.995 - 0.213 | 0.360 |
| Right precentral gyrus | -0.462 | 0.309 | -1.069 - 0.144 | 0.275 |
| Right precuneus | 0.039 | 0.305 | -0.559 - 0.637 | 0.912 |
| Right rostral anterior cingulate cortex | 0.612 | 0.312 | 0.000 - 1.225 | 0.161 |
| Right rostral middle frontal gyrus | -0.509 | 0.310 | -1.117 - 0.099 | 0.249 |
| Right superior frontal gyrus | -1.161 | 0.331 | -1.810 - -0.513 | 0.017* |
| Right superior parietal cortex | 0.381 | 0.308 | -0.222 - 0.985 | 0.365 |
| Right superior temporal gyrus | -1.171 | 0.331 | -1.820 - -0.521 | 0.017* |
| Right supramarginal gyrus | -0.410 | 0.308 | -1.014 - 0.195 | 0.354 |
| Right temporal pole | 0.043 | 0.305 | -0.555 - 0.641 | 0.912 |
| Right transverse temporal gyrus | -0.327 | 0.307 | -0.929 - 0.276 | 0.443 |
| Surface area right hemisphere | -0.851 | 0.319 | -1.477 - -0.226 | 0.114 |

HC: healthy controls; CI: confidence interval; FDR: false discovery rate; *FDR *p*-value < .05

**Table S2. ﻿**Results for thickness of each cortical region of interest for the 47,XXX subjects versus healthy controls comparison controlling for FSIQ.

|  | Cohen’s d  (47,XXX – HC) | Standard error | 95 % CI | FDR *p*-value |
| --- | --- | --- | --- | --- |
| Left banks superior temporal sulcus | 0.373 | 0.308 | -0.231 - 0.976 | 0.609 |
| Left caudal anterior cingulate cortex | -0.168 | 0.306 | -0.767 - 0.431 | 0.711 |
| Left caudal middle frontal gyrus | 0.349 | 0.308 | -0.254 - 0.952 | 0.609 |
| Left cuneus | 0.679 | 0.314 | 0.063 - 1.295 | 0.491 |
| Left entorhinal cortex | 0.462 | 0.309 | -0.144 - 1.068 | 0.609 |
| Left frontal pole | -0.251 | 0.306 | -0.852 - 0.349 | 0.660 |
| Left fusiform gyrus | 0.312 | 0.307 | -0.290 - 0.913 | 0.609 |
| Left inferior parietal cortex | 0.267 | 0.307 | -0.334 - 0.868 | 0.645 |
| Left inferior temporal cortex | 0.235 | 0.306 | -0.365 - 0.835 | 0.679 |
| Left insula | -0.565 | 0.311 | -1.176 - 0.045 | 0.499 |
| Left isthmus cingulate cortex | 0.660 | 0.314 | 0.046 - 1.275 | 0.491 |
| Left lateral occipital cortex | -0.044 | 0.305 | -0.642 - 0.554 | 0.925 |
| Left lateral orbitofrontal cortex | 0.146 | 0.306 | -0.453 - 0.744 | 0.718 |
| Left lingual gyrus | 0.198 | 0.306 | -0.402 - 0.797 | 0.711 |
| Left medial orbi-frontal cortex | 0.204 | 0.306 | -0.395 - 0.804 | 0.711 |
| Left middle temporal gyrus | 0.436 | 0.309 | -0.169 - 1.041 | 0.609 |
| Left paracentral lobule | 0.526 | 0.311 | -0.083 - 1.135 | 0.588 |
| Left parahippocampal gyrus | 0.382 | 0.308 | -0.221 - 0.986 | 0.609 |
| Left pars opercularis | 0.368 | 0.308 | -0.235 - 0.971 | 0.609 |
| Left pars orbitalis | 0.342 | 0.307 | -0.261 - 0.944 | 0.609 |
| Left pars triangularis | 0.423 | 0.309 | -0.182 - 1.028 | 0.609 |
| Left pericalcarine cortex | 0.332 | 0.307 | -0.270 - 0.934 | 0.609 |
| Left postcentral gyrus | 0.159 | 0.306 | -0.440 - 0.758 | 0.718 |
| Left posterior cingulate cortex | 0.199 | 0.306 | -0.400 - 0.799 | 0.711 |
| Left precentral gyrus | 0.464 | 0.309 | -0.142 - 1.071 | 0.609 |
| Left precuneus | 0.178 | 0.306 | -0.421 - 0.777 | 0.711 |
| Left rostral anterior cingulate cortex | 0.260 | 0.306 | -0.341 - 0.860 | 0.651 |
| Left rostral middle frontal gyrus | 0.177 | 0.306 | -0.422 - 0.776 | 0.711 |
| Left superior frontal gyrus | 0.470 | 0.309 | -0.136 - 1.077 | 0.609 |
| Left superior parietal cortex | 0.318 | 0.307 | -0.284 - 0.920 | 0.609 |
| Left superior temporal gyrus | 0.154 | 0.306 | -0.445 - 0.753 | 0.718 |
| Left supramarginal gyrus | 0.285 | 0.307 | -0.316 - 0.886 | 0.623 |
| Left temporal pole | 0.169 | 0.306 | -0.430 - 0.768 | 0.711 |
| Left transverse temporal gyrus | 0.169 | 0.306 | -0.430 - 0.768 | 0.711 |
| Average thickness left hemisphere | 0.291 | 0.307 | -0.310 - 0.892 | 0.623 |
| Right banks superior temporal sulcus | 0.319 | 0.307 | -0.283 - 0.921 | 0.609 |
| Right caudal anterior cingulate cortex | -0.416 | 0.309 | -1.021 - 0.189 | 0.609 |
| Right caudal middle frontal gyrus | 0.630 | 0.313 | 0.017 - 1.244 | 0.491 |
| Right cuneus | 0.373 | 0.308 | -0.230 - 0.976 | 0.609 |
| Right entorhinal cortex | 0.019 | 0.305 | -0.579 - 0.617 | 0.950 |
| Right frontal pole | 0.034 | 0.305 | -0.564 - 0.632 | 0.939 |
| Right fusiform gyrus | 0.319 | 0.307 | -0.283 - 0.921 | 0.609 |
| Right inferior parietal cortex | 0.169 | 0.306 | -0.430 - 0.768 | 0.711 |
| Right inferior temporal cortex | 0.293 | 0.307 | -0.308 - 0.894 | 0.623 |
| Right insula | -0.113 | 0.305 | -0.711 - 0.486 | 0.769 |
| Right isthmus cingulate cortex | 0.267 | 0.306 | -0.334 - 0.868 | 0.645 |
| Right lateral occipital cortex | 0.246 | 0.306 | -0.354 - 0.847 | 0.660 |
| Right lateral orbitofrontal cortex | -0.121 | 0.305 | -0.719 - 0.478 | 0.769 |
| Right lingual gyrus | 0.025 | 0.305 | -0.573 - 0.623 | 0.948 |
| Right medial orbitofrontal cortex | 0.207 | 0.306 | -0.392 - 0.807 | 0.711 |
| Right middle temporal gyrus | 0.224 | 0.306 | -0.375 - 0.824 | 0.694 |
| Right paracentral lobule | 0.583 | 0.312 | -0.028 - 1.194 | 0.491 |
| Right parahippocampal gyrus | 0.660 | 0.314 | 0.045 - 1.274 | 0.491 |
| Right pars opercularis | 0.323 | 0.307 | -0.279 - 0.925 | 0.609 |
| Right pars orbitalis | 0.598 | 0.312 | -0.014 - 1.209 | 0.491 |
| Right pars triangularis | 0.392 | 0.308 | -0.212 - 0.996 | 0.609 |
| Right pericalcarine cortex | 0.150 | 0.306 | -0.449 - 0.749 | 0.718 |
| Right postcentral gyrus | 0.585 | 0.312 | -0.026 - 1.196 | 0.491 |
| Right posterior cingulate cortex | 0.114 | 0.305 | -0.485 - 0.712 | 0.769 |
| Right precentral gyrus | 0.717 | 0.315 | 0.099 - 1.335 | 0.491 |
| Right precuneus | 0.336 | 0.307 | -0.267 - 0.938 | 0.609 |
| Right rostral anterior cingulate cortex | 0.511 | 0.310 | -0.097 - 1.119 | 0.594 |
| Right rostral middle frontal gyrus | 0.422 | 0.309 | -0.183 - 1.027 | 0.609 |
| Right superior frontal gyrus | 0.724 | 0.315 | 0.106 - 1.342 | 0.491 |
| Right superior parietal cortex | 0.314 | 0.307 | -0.288 - 0.915 | 0.609 |
| Right superior temporal gyrus | 0.166 | 0.306 | -0.433 - 0.765 | 0.711 |
| Right supramarginal gyrus | 0.332 | 0.307 | -0.271 - 0.934 | 0.609 |
| Right temporal pole | 0.287 | 0.307 | -0.314 - 0.889 | 0.623 |
| Right transverse temporal gyrus | -0.107 | 0.305 | -0.706 - 0.491 | 0.771 |
| Average thickness right hemisphere | 0.388 | 0.308 | -0.216 - 0.992 | 0.609 |

HC: healthy controls; CI: confidence interval; FDR: false discovery rate; *FDR *p*-value < .05

**Table S3. ﻿**Results for folding of each cortical region of interest for the 47,XXX subjects versus healthy controls comparison controlling for FSIQ and ICV.

|  | Cohen’s d  (47,XXX – HC) | Std. Err. | 95 % CI | FDR *p*-value |
| --- | --- | --- | --- | --- |
| Left banks superior temporal sulcus | 0.066 | 0.305 | -0.532 - 0.664 | 0.969 |
| Left caudal anterior cingulate cortex | -0.316 | 0.307 | -0.918 - 0.286 | 0.510 |
| Left caudal middle frontal gyrus | -0.104 | 0.305 | -0.702 - 0.494 | 0.892 |
| Left cuneus | -0.820 | 0.318 | -1.444 - -0.196 | 0.089 |
| Left entorhinal cortex | -0.764 | 0.317 | -1.385 - -0.144 | 0.114 |
| Left frontal pole | 0.001 | 0.305 | -0.596 - 0.599 | 0.996 |
| Left fusiform gyrus | -0.360 | 0.308 | -0.963 - 0.243 | 0.476 |
| Left inferior parietal cortex | 0.016 | 0.305 | -0.582 - 0.614 | 0.985 |
| Left inferior temporal cortex | 0.166 | 0.306 | -0.433 - 0.765 | 0.771 |
| Left insula | -0.875 | 0.320 | -1.502 - -0.248 | 0.084 |
| Left isthmus cingulate cortex | -0.706 | 0.315 | -1.323 - -0.089 | 0.147 |
| Left lateral occipital cortex | 0.170 | 0.306 | -0.430 - 0.769 | 0.771 |
| Left lateral orbitofrontal cortex | -0.581 | 0.312 | -1.192 - 0.030 | 0.198 |
| Left lingual gyrus | -0.870 | 0.320 | -1.497 - -0.243 | 0.084 |
| Left medial orbi-frontal cortex | -0.315 | 0.307 | -0.917 - 0.286 | 0.510 |
| Left middle temporal gyrus | 0.018 | 0.305 | -0.580 - 0.616 | 0.985 |
| Left paracentral lobule | -0.360 | 0.308 | -0.963 - 0.243 | 0.476 |
| Left parahippocampal gyrus | -1.051 | 0.326 | -1.691 - -0.412 | 0.084 |
| Left pars opercularis | -0.654 | 0.314 | -1.268 - -0.039 | 0.157 |
| Left pars orbitalis | -0.696 | 0.315 | -1.313 - -0.079 | 0.147 |
| Left pars triangularis | -0.855 | 0.319 | -1.481 - -0.229 | 0.084 |
| Left pericalcarine cortex | -0.957 | 0.323 | -1.590 - -0.324 | 0.084 |
| Left postcentral gyrus | -0.184 | 0.306 | -0.784 - 0.415 | 0.746 |
| Left posterior cingulate cortex | -0.298 | 0.307 | -0.900 - 0.303 | 0.541 |
| Left precentral gyrus | -0.228 | 0.306 | -0.827 - 0.372 | 0.666 |
| Left precuneus | -0.681 | 0.314 | -1.297 - -0.065 | 0.153 |
| Left rostral anterior cingulate cortex | -0.554 | 0.311 | -1.164 - 0.055 | 0.208 |
| Left rostral middle frontal gyrus | -0.322 | 0.307 | -0.924 - 0.280 | 0.510 |
| Left superior frontal gyrus | -0.270 | 0.307 | -0.871 - 0.330 | 0.589 |
| Left superior parietal cortex | 0.022 | 0.305 | -0.575 - 0.620 | 0.985 |
| Left superior temporal gyrus | -0.705 | 0.315 | -1.322 - -0.087 | 0.147 |
| Left supramarginal gyrus | -0.099 | 0.305 | -0.697 - 0.500 | 0.892 |
| Left temporal pole | -0.366 | 0.308 | -0.969 - 0.237 | 0.476 |
| Left transverse temporal gyrus | -0.650 | 0.313 | -1.264 - -0.035 | 0.157 |
| Right banks superior temporal sulcus | 0.032 | 0.305 | -0.566 - 0.630 | 0.985 |
| Right caudal anterior cingulate cortex | -0.215 | 0.306 | -0.815 - 0.385 | 0.688 |
| Right caudal middle frontal gyrus | -0.348 | 0.307 | -0.951 - 0.255 | 0.480 |
| Right cuneus | -0.619 | 0.313 | -1.232 - -0.006 | 0.167 |
| Right entorhinal cortex | -0.042 | 0.305 | -0.640 - 0.556 | 0.985 |
| Right frontal pole | -0.252 | 0.306 | -0.853 - 0.348 | 0.611 |
| Right fusiform gyrus | -0.416 | 0.309 | -1.021 - 0.189 | 0.409 |
| Right inferior parietal cortex | -0.135 | 0.305 | -0.734 - 0.463 | 0.846 |
| Right inferior temporal cortex | 0.103 | 0.305 | -0.496 - 0.701 | 0.892 |
| Right insula | -0.642 | 0.313 | -1.255 - -0.028 | 0.157 |
| Right isthmus cingulate cortex | -0.641 | 0.313 | -1.255 - -0.027 | 0.157 |
| Right lateral occipital cortex | -0.260 | 0.306 | -0.860 - 0.341 | 0.604 |
| Right lateral orbitofrontal cortex | -0.353 | 0.308 | -0.956 - 0.250 | 0.480 |
| Right lingual gyrus | -0.560 | 0.311 | -1.170 - 0.050 | 0.208 |
| Right medial orbi-frontal cortex | 0.012 | 0.305 | -0.586 - 0.609 | 0.985 |
| Right middle temporal gyrus | 0.113 | 0.305 | -0.485 - 0.712 | 0.892 |
| Right paracentral lobule | -0.535 | 0.311 | -1.144 - 0.074 | 0.213 |
| Right parahippocampal gyrus | -0.608 | 0.312 | -1.220 - 0.004 | 0.173 |
| Right pars opercularis | -0.844 | 0.319 | -1.469 - -0.218 | 0.084 |
| Right pars orbitalis | -0.062 | 0.305 | -0.660 - 0.536 | 0.969 |
| Right pars triangularis | -0.567 | 0.311 | -1.178 - 0.043 | 0.208 |
| Right pericalcarine cortex | -0.635 | 0.313 | -1.249 - -0.022 | 0.157 |
| Right postcentral gyrus | -0.293 | 0.307 | -0.895 - 0.308 | 0.541 |
| Right posterior cingulate cortex | -0.542 | 0.311 | -1.152 - 0.067 | 0.210 |
| Right precentral gyrus | -0.376 | 0.308 | -0.980 - 0.227 | 0.476 |
| Right precuneus | -0.762 | 0.316 | -1.382 - -0.142 | 0.114 |
| Right rostral anterior cingulate cortex | -0.322 | 0.307 | -0.924 - 0.280 | 0.510 |
| Right rostral middle frontal gyrus | -0.549 | 0.311 | -1.159 - 0.060 | 0.208 |
| Right superior frontal gyrus | -0.466 | 0.309 | -1.072 - 0.141 | 0.316 |
| Right superior parietal cortex | -0.012 | 0.305 | -0.610 - 0.586 | 0.985 |
| Right superior temporal gyrus | -0.374 | 0.308 | -0.978 - 0.229 | 0.476 |
| Right supramarginal gyrus | -0.202 | 0.306 | -0.802 - 0.398 | 0.710 |
| Right temporal pole | -0.034 | 0.305 | -0.632 - 0.564 | 0.985 |
| Right transverse temporal gyrus | -0.847 | 0.319 | -1.472 - -0.221 | 0.084 |

HC: healthy controls; CI: confidence interval; FDR: false discovery rate; *FDR P-value < .05

**Table S4. ﻿**Correlation between thickness of each cortical region of interest and social cognition in 47,XXX subjects.

|  | Pearson correlation coefficient | FDR *p*-value |
| --- | --- | --- |
| Left banks superior temporal sulcus | 0.148 | 0.651 |
| Left caudal anterior cingulate cortex | -0.618 | 0.049* |
| Left caudal middle frontal gyrus | 0.427 | 0.178 |
| Left cuneus | 0.410 | 0.189 |
| Left entorhinal cortex | 0.304 | 0.337 |
| Left frontal pole | 0.102 | 0.747 |
| Left fusiform gyrus | 0.380 | 0.224 |
| Left inferior parietal cortex | 0.506 | 0.149 |
| Left inferior temporal cortex | 0.432 | 0.178 |
| Left insula | 0.144 | 0.652 |
| Left isthmus cingulate cortex | -0.026 | 0.926 |
| Left lateral occipital cortex | 0.639 | 0.042* |
| Left lateral orbitofrontal cortex | 0.405 | 0.192 |
| Left lingual gyrus | 0.385 | 0.220 |
| Left medial orbitofrontal cortex | 0.465 | 0.158 |
| Left middle temporal gyrus | 0.322 | 0.319 |
| Left paracentral lobule | 0.423 | 0.178 |
| Left parahippocampal gyrus | -0.226 | 0.472 |
| Left pars opercularis | 0.271 | 0.366 |
| Left pars orbitalis | 0.289 | 0.365 |
| Left pars triangularis | 0.318 | 0.319 |
| Left pericalcarine cortex | 0.646 | 0.042* |
| Left postcentral gyrus | -0.125 | 0.688 |
| Left posterior cingulate cortex | -0.459 | 0.158 |
| Left precentral gyrus | 0.273 | 0.366 |
| Left precuneus | 0.346 | 0.272 |
| Left rostral anterior cingulate cortex | 0.167 | 0.609 |
| Left rostral middle frontal gyrus | 0.279 | 0.366 |
| Left superior frontal gyrus | 0.272 | 0.366 |
| Left superior parietal cortex | 0.483 | 0.158 |
| Left superior temporal gyrus | 0.049 | 0.871 |
| Left supramarginal gyrus | 0.349 | 0.272 |
| Left temporal pole | 0.310 | 0.333 |
| Left transverse temporal gyrus | 0.168 | 0.609 |
| Average thickness left hemisphere | 0.436 | 0.178 |
| Right banks superior temporal sulcus | 0.546 | 0.121 |
| Right caudal anterior cingulate cortex | -0.207 | 0.516 |
| Right caudal middle frontal gyrus | 0.090 | 0.751 |
| Right cuneus | 0.548 | 0.121 |
| Right entorhinal cortex | 0.091 | 0.751 |
| Right frontal pole | -0.006 | 0.978 |
| Right fusiform gyrus | 0.513 | 0.149 |
| Right inferior parietal cortex | 0.470 | 0.158 |
| Right inferior temporal cortex | 0.411 | 0.189 |
| Right insula | 0.091 | 0.751 |
| Right isthmus cingulate cortex | 0.161 | 0.620 |
| Right lateral occipital cortex | 0.518 | 0.149 |
| Right lateral orbitofrontal cortex | 0.343 | 0.272 |
| Right lingual gyrus | 0.386 | 0.220 |
| Right medial orbitofrontal cortex | 0.364 | 0.245 |
| Right middle temporal gyrus | 0.271 | 0.366 |
| Right paracentral lobule | 0.425 | 0.178 |
| Right parahippocampal gyrus | -0.137 | 0.656 |
| Right pars opercularis | 0.116 | 0.706 |
| Right pars orbitalis | 0.463 | 0.158 |
| Right pars triangularis | 0.231 | 0.469 |
| Right pericalcarine cortex | 0.474 | 0.158 |
| Right postcentral gyrus | -0.039 | 0.892 |
| Right posterior cingulate cortex | 0.081 | 0.771 |
| Right precentral gyrus | 0.278 | 0.366 |
| Right precuneus | 0.464 | 0.158 |
| Right rostral anterior cingulate cortex | 0.142 | 0.652 |
| Right rostral middle frontal gyrus | 0.211 | 0.514 |
| Right superior frontal gyrus | 0.263 | 0.379 |
| Right superior parietal cortex | 0.641 | 0.042* |
| Right superior temporal gyrus | 0.365 | 0.245 |
| Right supramarginal gyrus | 0.202 | 0.522 |
| Right temporal pole | 0.174 | 0.608 |
| Right transverse temporal gyrus | 0.302 | 0.337 |
| Average thickness right hemisphere | 0.440 | 0.178 |

FDR: false discovery rate; *FDR *p*-value < .05
